# 'Rapunzel syndrome' trichobezoar in a 7-year-old girl: a case report

**DOI:** 10.1186/1757-1626-1-205

**Published:** 2008-10-02

**Authors:** Ali H Al Wadan, Hamed Al Kaff, Jamila Al Senabani, Azan S Al Saadi

**Affiliations:** 1 Kwait University Hospital, Sana'a medical SchooL, Wadi Dhaher Road, P.O. Box 1247, Sana'a, Yemen

## Abstract

**Background:**

Rapunzel syndrome is a rare type of trichobezoar with an extension of the hair into the small bowel. Clinical presentation is deceptive and vague ranging from abdominal mass to gastrointestinal symptoms.

**Case presentation:**

We present a 7 years old girl with Rapunzel syndrome, where the trichobezoar was not suspected at all especially with negative history of trichophagia. In majority of the cases the diagnosis was made very late in the history of the disease, at a stage where surgery is the only cure for this syndrome.

**Conclusion:**

In the paediatric age group with a long history of gastrointestinal symptom, endoscopy is a diagnostic as well as a therapeutic modality and may reduce surgery in trichobezoars.

## Background

Bezoar is a tightly packed collection of undigested material that is unable to exit the stomach, Most bezoars are of indigestible organic matter such as hair-trichobezoars; or vegetable and fruit the – phytobezoars; or a combination of both but other rare substances has been also been described in literature. Trichobezoars, commonly occur in patients with psychiatric disturbances who chew and swallow their own hair. Only 50% will have history of trichophagia. Trichobezoars have been described in literature and they comprise 55% of all bezoars. In very rare cases the Rapunzel Syndrome hair extends through the pylorus into the small bowel causing symptom and sign of partial or complete gastric outlet obstruction [1].

## Case presentation

7 year old girl was referred to our paediatric clinic in 2007, with a history of abdominal pain, distension, weight loss and attacks of vomiting which was not persistent but it comes after a meal and fluid intake. This history was on and off for almost one year. She was treated in different primary health care as a case of gastroenteritis and parasitic infestation. Despite the treatment she was receiving there was no improvement in her condition, in fact she was getting worse.

In the paediatric clinic, abdominal palpation revealed an oblong mobile well-defined mass occupying the upper half of the abdomen, the mass was not tender and was firm in consistency. Upper Gastrointestinal endoscopy revealed a Trichobezoar occupying almost the whole gastric cavity, an attempt to remove it by foreign body forceps failed and the forceps was barley pulling few fibres of this huge ball of hair.

**Figure 1:**
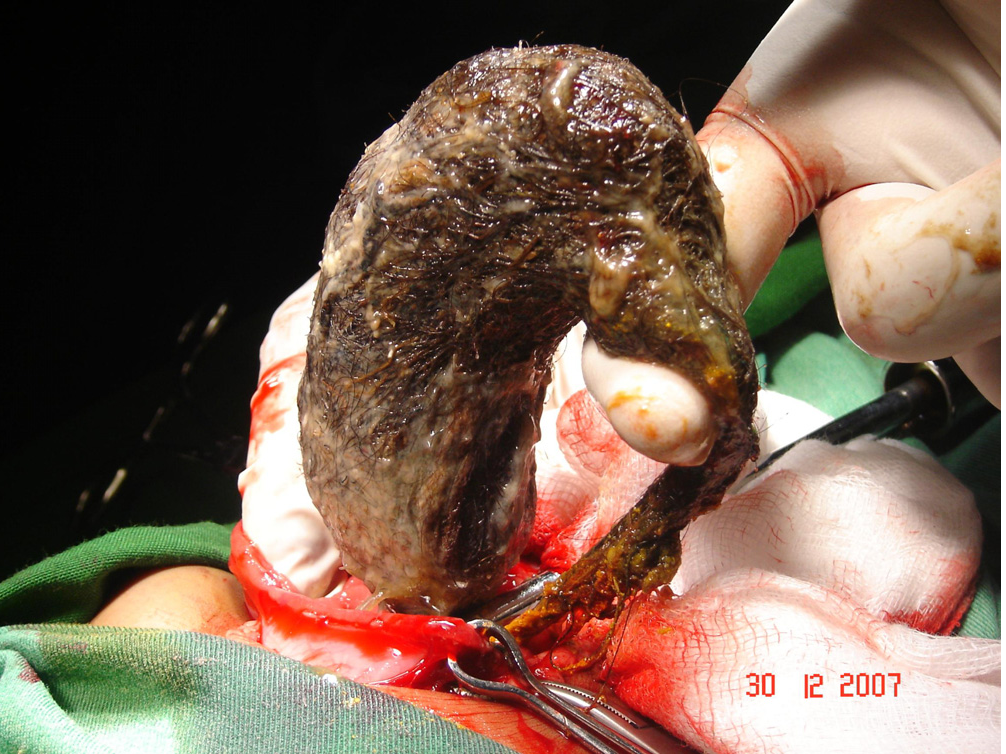
Trichobezoar extracted from the stomach following gastrotomy.

**Figure 2:**
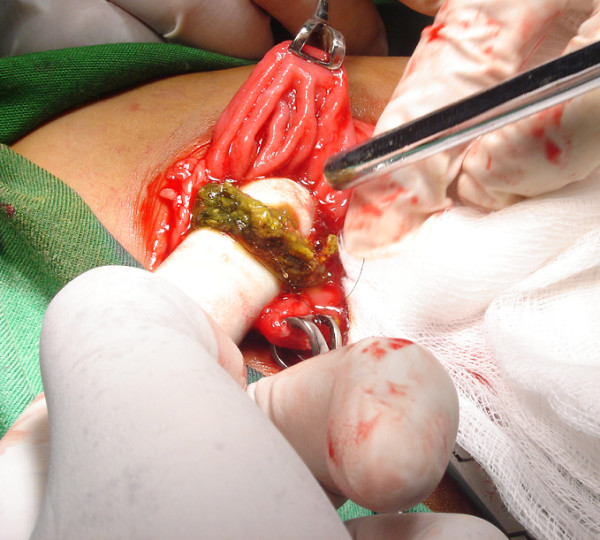
The extension of the hair into the small bowel through entrotomy.

**Figure 3:**
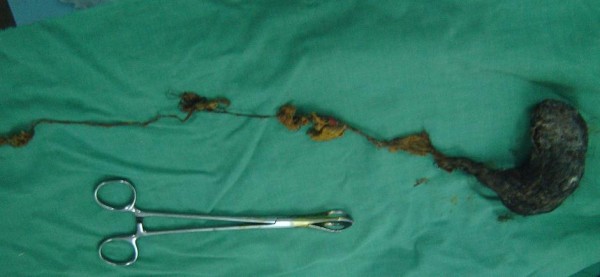
The whole hair extracted with its tail which was extracted from the small bowel.

The patient was referred to Surgery, and through upper mid line incision the stomach was opened (Gastrotomy) between two Vicryl stay suture. A huge Trichobezoar was identified which took the shape of the stomach (Figure 1, 2). There was a long tail of hair extending through the pylorus into the small bowel. By this feature the diagnosis was clear of a Rapunzel syndrome. A distal enterotomy was performed (Figure 3) and the remaining part was dislodged gently. Both the opening in the gastric wall and small bowel was closed with continuous Vicryl. The patient had an uneventful postoperative course and was discharged after six days. The parents were advised to visit pediatric psychiatry for follow up.

## Discussion

The common presentation of trichobezoar is in young females usually with an underlying psychiatric disorder. In our case the presentation is in a very young age with hair extending down to the small bowel, causing symptoms, which could mimic gastrointestinal infections and infestation especially in endemic areas. Rapunzel Syndrome is a rare form of trichobezoar. It is named after a tale written in 1812 by the Brothers Grimm about a young maiden, Rapunzel, with long hair who lowered her hair to the ground from a castle, which was a prison tower to permit her young prince to climb up to her window and rescue her.

In recent years more and more cases has been described [2]. This syndrome was originally described by Vaughan et al. in 1968 [3]. The commonly accepted definition is that of a gastric trichobezoar with a tail extending to the jejunum, ileum or the ileocecal junction.

Majority of cases of trichobezoar present late, due to the low index of suspicion by the physician. Of 131 collected cases of trichobezoar, a palpable abdominal mass was present in (87.7%), abdominal pain (70.2%), nausea and vomiling (64.9%), weakness and weight loss (38.1%), constipation or diarrhoea (32%) and haematemesis (6.1%). The laboratory investigations revealed low haemogiobin in about 62% (average) [4].

The complications of Rapunzel syndrome ranges from attacks of incomplete pyloric obstruction to complete obstruction of the bowel to perforation to peritonitis and mortality [5-7].

Patient with Trichotillomania (a psychological condition that involves strong urges to pull hair), around 30% will engage in trichophagia, and of these, only 1% will go on to eat their hair to the extent requiring surgical removal [8]. Less than half of the patients give a history of trichophagia. There has been few cases of recurrence following successful surgery [9].

Endoscopy is diagnostic, in almost all cases while Ultrasound has not much to offer as a diagnostic tool. CT scan with contrast will delineate the extension of trichobezoar.

In the early stages endoscopic removal is not with out risk of bowel perforation and should be resolved for small Trichobezoars only [10]. Other methods including the use of laser ignited mini-explosive technique were used successfully [11]. Laparoscopy has been also used with limited success. Open surgery still remains the corner stone of large Trichobezoar removal especially if it has an extension into the bowel, which might be missed with other methods of treatment.

## Conclusion

A long history of gastrointestinal problem, in a pediatric age group with history of trichophagia, early endoscopy is recommended. All patients with Trichobezoar should be referred for psychiatric evaluation after surgery to avoid recurrence.

## Competing interests

The authors declare that they have no competing interests.

## Consent

Written informed consent was obtained from the parents for publication of this case report and accompanying images. A copy of the written consent is available for review by the Editor-in-Chief of this journal.

## Authors' contributions

The authors were involved in patient management or writing of the manuscript.

AW and JS performed the surgery and the follow up of the patient, HK diagnosed the case, did endoscopy and refered the patient for surgery. AS did the workup preoperatively and was the Major contributor in writing the manuscript. All authors read and approved the final manuscript.
